# Revealing the relative importance among plant species, slope positions, and soil types on rhizosphere microbial communities in northern tropical karst and non-karst seasonal rainforests of China

**DOI:** 10.3389/fmicb.2023.1103550

**Published:** 2023-04-17

**Authors:** Xingming Zhang, Bin Wang, Ting Chen, Yili Guo, Xiankun Li

**Affiliations:** ^1^Guangxi Key Laboratory of Plant Conservation and Restoration Ecology in Karst Terrain, Guangxi Institute of Botany, Guangxi Zhuang Autonomous Region and Chinese Academy of Sciences, Guilin, China; ^2^College of Urban Construction, Wuchang Shouyi University, Wuhan, China; ^3^Nonggang Karst Ecosystem Observation and Research Station of Guangxi, Chongzuo, Guangxi, China

**Keywords:** rhizosphere microbial community, northern tropical seasonal rainforest, soil types, plant species, slope positions, relative importance

## Abstract

Rhizosphere microbes have an extremely close relationship with plants and the study on the relationship between rhizosphere microorganisms and their influencing factors is conducive to the protection of vegetation and the maintenance of biodiversity. Here we investigated how plant species, slope positions and soil types affect the rhizosphere microbial community. Slope positions and soil types were collected from northern tropical karst and non-karst seasonal rainforests. The results indicated that soil types played a predominant role in the development of rhizosphere microbial communities (28.3% of separate contribution rate), more than plant species identity (10.9% of separate contribution rate) and slope position (3.5% of separate contribution rate). Notably, environmental factors closely related to soil properties were the major influence factors that controlling the rhizosphere bacterial community structure in the northern tropical seasonal rainforest, especially pH. Additionally, plant species also influenced the rhizosphere bacterial community. In low nitrogen content soil environments, rhizosphere biomarkers of dominant plant species were often nitrogen-fixing strains. It suggested that plants might have a selective adaptation mechanism to rhizosphere microorganisms to obtain the advantages of nutrient supply. Overall, soil types exerted the biggest influence on rhizosphere microbial community structure, followed by plant species and finally slope positions.

## 1. Introduction

The rhizosphere, a small volume of soil surrounding and influenced by plant roots, is the most dynamic habitat and is regarded as the most important zone and one of the most complicated ecosystems on Earth (Hinsinger et al., 2009; Mendes et al., 2013). Rhizosphere microbes have an extremely close relationship with plants. Due to this, they are thought to be the second genome of the plant (Berendsen et al., 2012; Saleem et al., 2016). The development of plants is subjected to internal signals, depending on the provided mineral nutrients through the rhizosphere (from soil to the roots) (Dakora and Phillips, 2002). Thus, the rhizosphere environment provides unique and fundamental points of plant-microbial symbioses (Hao et al., 2016). Bacteria, as the primary component of rhizosphere microbiome food webs, play an important role in the nutrient cycle in the rhizosphere. This is the key to the isolation of prospective biotransformation strains, agricultural practices, and potential biocontrol strains (Maurhofer et al., 2004; Hao et al., 2008, 2009). Despite the importance of rhizosphere microbes, the relative importance of various factors and combined effects affecting the structure of rhizosphere microbial communities are still poorly understood (Igwe and Vannette, 2019).

Plants can produce an effect on the rhizosphere microbial community structure and activity. This is based on their behavior of secreting photosynthetically fixed carbon in the rhizosphere (Berendsen et al., 2012). Furthermore, plant rhizosphere exudates can also play a regulating role in the type and composition of microorganisms. For example, studies on potato rhizosphere exudates have shown that the photosynthetic products of rhizosphere exudates are different in different varieties of potatoes and different growth stages, which in turn have a greater impact on the rhizosphere microbial community (Gschwendtner et al., 2011). Plants also affect rhizosphere microorganisms through the generation of biological signals. Studies have shown that when the leaves of *Arabidopsis thaliana* are infected, they conduct biological signals to their roots and induce the release of malic acid, and the aggregation of malic acid in the rhizosphere promotes the aggregation of beneficial bacteria, thereby enhancing the disease resistance of *Arabidopsis thaliana* (Lakshmanan et al., 2012). In addition to plant traits, such as plant species characteristics and rhizosphere secretions, which have significant influences on rhizosphere microbial community structure, other investigations have shown that the physicochemical properties of rhizosphere soil also play a key decisive role.

Indeed, rhizosphere microbes are highly dependent on soil abiotic environmental factors. Previous studies have investigated the relationships between soil biogeochemical properties and microbial community, such as ammonium nitrogen (AN), soil organic matter, available K and N, pH, and nutrient content (Gu et al., 2018; Nan et al., 2020). Some scholars studied the effects of three types of slope aspects on soil bacteria and arbuscular mycorrhizal fungal communities in a boreal forest of the Greater Khingan Mountains and found that soil pH and substratum shrub biomass were significantly correlated with bacterial communities, and soil available phosphorus and shrub biomass were significantly correlated with arbuscular mycorrhizal fungal communities. In addition, they found that slope aspects affected bacterial and AMF communities, mediated by aspect-induced changes in plant community and soil chemical properties (Chu et al., 2016). Thus, in addition to the plant traits, such as plant species characteristics and rhizosphere secretions, soil biogeochemical properties and slope aspects also have a considerable impact on the rhizosphere microbial community, a better understanding of the relative importance of these three factors and their combined effect is essential. Although some of the previous studies have addressed the effect of plant species or rhizosphere biogeochemical properties on rhizosphere microbial community composition and diversity, they have mostly emphasized crop plants with cultivation-based approaches in long-term treated soils (Pii et al., 2016; Leff et al., 2018). The rhizosphere microbial community is very complicated, so only a few studies based on the culture-independent approach have focused on natural plant species (Hao et al., 2016). Recently, with the introduction of PCR-based and high-throughput sequencing technologies, a new molecular view of the microbial world is rendered on the ground that it has improved the feature of natural microbial communities in complex environments such as rhizosphere soil (Lynch and Neufeld, 2015).

Northern tropical seasonal rain forest includes karst seasonal rain forest and non-karst seasonal rain forest. It is a unique and huge treasure pool of biological diversity in the southwest karst area of China and one of the global biodiversity hotspots (Myers et al., 2000). The same period of rain and heat climatic conditions, the low soil formation rate, and other natural processes in this region have given rise to serious soil erosion in the karst area as well as difficulty in vegetation restoration (Jiang et al., 2014). Since there is no anthropogenic disturbance for over 100 years, this reserve has the most typical and aboriginal karst seasonal rainforest in China (Guo et al., 2018). The study of the northern tropical karst seasonal rainforest could help deepen the conservation and maintenance of the seasonal rainforest biodiversity. However, as to the northern tropical seasonal rainforest, there is very limited knowledge on the relationship of the rhizosphere microbial community composition with diversity with the dominant species in virgin forests. Moreover, it is still unclear about the combined effects and relative importance of various factors that affect the structure of the rhizosphere microbial community (Liu et al., 2020).

In this study, based on the high-throughput sequencing technology and multiple regression analysis, the relative importance and effects of plant species, slope positions, and soil types on the rhizosphere microbial community of the northern tropical seasonal rainforest were studied. This research would enhance the conservation and maintenance of the seasonal rainforest biodiversity in the southwest karst area of China.

## 2. Materials and methods

### 2.1. Sample site

**Figure 1:**
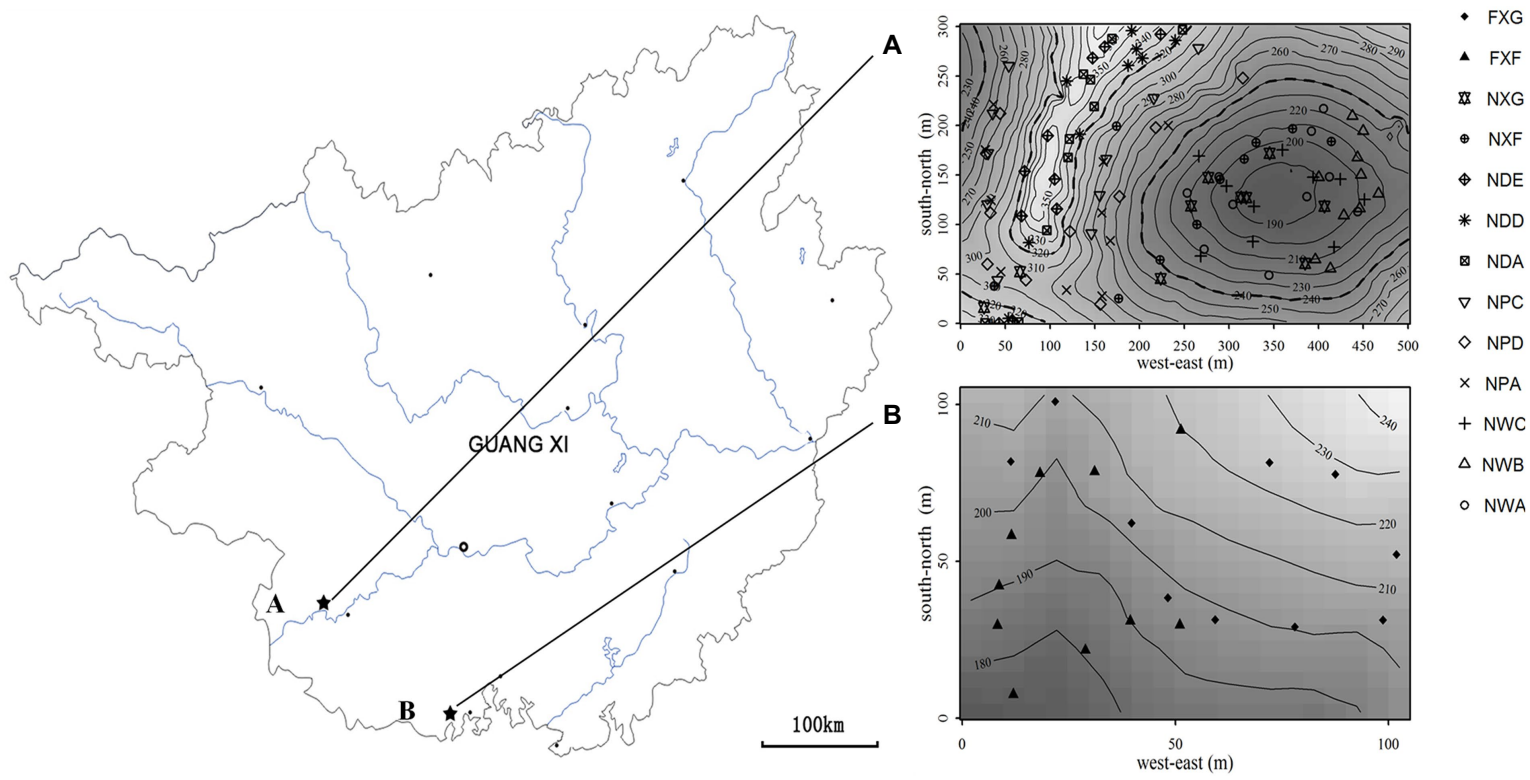
Locations of the sampling sites in two plots in Guangxi province (R software (version 3.3.0) was employed to produce Maps). A: Nonggang northern tropical karst seasonal rainforest dynamics plot; **(A)**: sampling points of the Nonggang plot. B: Fangcheng northern tropical seasonal rainforest dynamics plot; **(B)**: sampling points of the Fangcheng plot.

The northern tropical seasonal rainforest of China is a zonal vegetation type of northern tropics in south Guangxi (Wang et al., 2001), which include karst and non-karst seasonal rainforest in the acid soil region. At the end of August 2016, 48 soil samples, including 39 rhizosphere soil samples and nine non-rhizosphere soil samples, were collected from the northern tropical seasonal rainforest of China at two sites in Guangxi Province: (a) Nonggang Northern Tropical Karst Seasonal Rainforest Dynamics Plot (106.95°E, 22.43°N, 15 ha, Figure 1) and (b) Fangcheng Northern Tropical Seasonal Rainforest Dynamics Plot (108.136°E, 21.775°N, 1 ha, Figure 1). Site A was located inside Nongang National Nature Reserve, which was typical karst peak-cluster depression landform with karst lime soil (Wang et al., 2014). Sample site B was located inside Fangcheng *Camellia nitidissima Chi* National Nature Reserve, which was a non-karst area. The landform type was coastal hills and mesas (Huang et al., 2013), and the soil type was laterite and brick red soil formed from granite, sand shale, mudstone, and conglomerate (Huang, 2000).

### 2.2. Experimental design

Sample site A was divided into the following three slope positions: around the shoulder, the back slope, and the toe slope. The rhizosphere soil was collected from the dominant species and common species in each slope position. Two common species of rhizosphere soil were gathered at both sites (Table 1). The experimental groups were as follows: (1) NWA, NWB, and NWC, which were sampled around the toe slope in site A and represented the rhizosphere of *Sterculia monosperma*, *Saraca dives*, and *Catunaregam spinosa*, respectively; (2) NPA, NPD, and NPC, which were sampled in the back slope and represented the rhizosphere of *Sterculia monosperma*, *Excentrodendron tonkinensis*, and *Catunaregam spinosa*, respectively; (3) NDA, NDD, and NDE, which were sampled around the shoulder and represented the rhizosphere of *Sterculia monosperma*, *Excentrodendron tonkinensis*, and *Boniodendron minius*, respectively; (4) NXF and NXG, which were sampled from site A and represented the rhizosphere of *Wendlandia uvariifolia* and *Bridelia balansae*; (5) FXF and FXG, which were sampled from site B and represented the rhizosphere of *Wendlandia uvariifolia* and *Bridelia balansae*; (6) NWK, NPK, and NDK represented non-rhizosphere soil samples and were collected for each slope position in site A as control groups.

**Table 1:** Samples description.

Groups	Samples sites	Slope positions	Soil types	Cover plants
NWA	Nonggang Northern Tropical Karst Seasonal Rainforest Dynamics Plot	Toe slope	Lime soil	*Sterculia monosperma*
NWB	Nonggang Northern Tropical Karst Seasonal Rainforest Dynamics Plot	Toe slope	Lime soil	*Saraca dives*
NWC	Nonggang Northern Tropical Karst Seasonal Rainforest Dynamics Plot	Toe slope	Lime soil	*Catunaregam spinosa*
NWK	Nonggang Northern Tropical Karst Seasonal Rainforest Dynamics Plot	Toe slope	Lime soil	none
NPA	Nonggang Northern Tropical Karst Seasonal Rainforest Dynamics Plot	Back slope	Lime soil	*Sterculia monosperma*
NPD	Nonggang Northern Tropical Karst Seasonal Rainforest Dynamics Plot	Back slope	Lime soil	*Excentrodendron tonkinense*
NPC	Nonggang Northern Tropical Karst Seasonal Rainforest Dynamics Plot	Back slope	Lime soil	*Catunaregam spinosa*
NPK	Nonggang Northern Tropical Karst Seasonal Rainforest Dynamics Plot	Back slope	Lime soil	none
NDA	Nonggang Northern Tropical Karst Seasonal Rainforest Dynamics Plot	Shoulder	Lime soil	*Sterculia monosperma*
NDD	Nonggang Northern Tropical Karst Seasonal Rainforest Dynamics Plot	Shoulder	Lime soil	*Excentrodendron tonkinense*
NDE	Nonggang Northern Tropical Karst Seasonal Rainforest Dynamics Plot	Shoulder	Lime soil	*Boniodendron minius*
NDK	Nonggang Northern Tropical Karst Seasonal Rainforest Dynamics Plot	Shoulder	Lime soil	none
NXF	Nonggang Northern Tropical Karst Seasonal Rainforest Dynamics Plot	Toe slope	Lime soil	*Wendlandia uvariifolia*
NXG	Nonggang Northern Tropical Karst Seasonal Rainforest Dynamics Plot	Toe slope	Lime soil	*Bridelia balansae*
FXF	Fangcheng Northern Tropical Seasonal Rainforest Dynamics Plot	Back slope	Laterite and brick red soil	*Wendlandia uvariifolia*
FXG	Fangcheng Northern Tropical Seasonal Rainforest Dynamics Plot	Toe slope	Laterite and brick red soil	*Bridelia balansae*

### 2.3. Sample collection and DNA extraction

The plants are aged 8–20 years. Roots, rock, and large chunks of non-rooted soil were moved away. The collection of rhizosphere samples was made in triplicate from 10 individual plants of each species within the field of 15 ha and 1 ha (Figure 1). The control groups at each slope position were sampled at 10 spots in triplicate using the “S”-shaped sampling method. Rhizosphere samples and control samples were divided into two parts, one part for the analysis of biogeochemical properties and the other stored at −80°C for nucleic acid analyses. Genomic DNA from soil samples was extracted by applying the PowerSoil DNA Isolation Kit (MoBio Laboratories, Carlsbad, CA). Agarose gel electrophoresis was applied to examine DNA, and the NanoDrop spectrophotometer was employed to check its purity.

### 2.4. Analysis method

From each sampling site, a composite sample was obtained by gathering and mixing three soil samples. After oven drying part of the samples at 105°C to a constant weight, it was weighed for the determination of moisture content (MC). For pH, soil organic carbon (SOC), total nitrogen (TN), total phosphorus (TP), hydrolyzable nitrogen (HN), and available phosphate (AP) measurements, part of the soil samples were manually separated to visually remove stones, plant roots, and litter, and then sieved through a 0.25 mm mesh. An FE20 pH meter (Mettler Toledo, Shanghai, China) was adopted to measure soil pH values at a soil-to-water (deionized) ratio of 1: 2.5. The K_2_Cr_2_O_7_/H_2_SO_4_ oxidation method was employed to determine SOC concentrations. The Kjeldahl method was employed to determine TN concentrations, and the sodium hydroxide (NaOH) fusion and Mo–Sb colorimetric methods were used to measure TP concentrations (Jiang et al., 2017). HN was determined using the Illinois Soil N Test (ISNT) method (Roberts et al., 2009). AP was determined by the NaHCO_3_ procedure and heteropoly molybdenum blue method (Mussa et al., 2009). Calcium (Ca) and magnesium (Mg) were determined based on chemical methods (Lu, 2000). Each experiment was conducted three times in parallel, and the results are indicated in Table 2.

**Table 2:** Mean biogeochemical properties of samples collected in two plots.

Sample sites	Moisture content (%)	Total nitrogen (g kg^−1^)	Total phosphorus (g kg^−1^)	Organic Carbon (%)	Hydrolyzable nitrogen (mg kg^−1^)	Available phosphate (mg kg^−1^)	pH water (1:2.5)	Calcium (g kg^−1^)	Magnesium (g kg^−1^)
NWA	39.37 ± 0.13	14.64 ± 0.03	2.87 ± 0.02	6.99 ± 0.03	234.5 ± 3.97	5.16 ± 0.10	7.99 ± 0.05	18.89 ± 0.14	10.24 ± 0.03
NWB	39.91 ± 0.60	13.86 ± 0.12	1.88 ± 0.03	5.95 ± 0.04	254.1 ± 8.48	22.23 ± 0.06	7.69 ± 0.04	17.93 ± 0.03	12.07 ± 0.05
NWC	41.32 ± 0.99	14.51 ± 0.07	3.37 ± 0.09	7.19 ± 0.06	368.2 ± 3.80	17.91 ± 0.09	7.83 ± 0.02	20.98 ± 0.08	11.51 ± 0.08
NWK	37.48 ± 0.10	13.47 ± 0.02	1.88 ± 0.05	5.07 ± 0.03	273.0 ± 6.37	18.90 ± 0.11	7.99 ± 0.03	18.96 ± 0.03	9.68 ± 0.04
NPA	38.08 ± 0.14	15.94 ± 0.06	0.40 ± 0.05	5.87 ± 0.02	287.0 ± 1.68	5.14 ± 0.09	8.07 ± 0.01	20.12 ± 0.10	6.52 ± 0.05
NPD	29.67 ± 0.05	13.60 ± 0.09	0.40 ± 0.01	5.78 ± 0.01	203.0 ± 2.76	0.83 ± 0.08	8.05 ± 0.02	17.98 ± 0.08	10.90 ± 0.20
NPC	33.82 ± 0.08	17.75 ± 0.10	0.89 ± 0.02	6.50 ± 0.01	303.8 ± 7.18	6.17 ± 0.11	8.59 ± 0.03	15.87 ± 0.03	6.01 ± 0.06
NPK	31.94 ± 0.27	17.62 ± 0.06	0.89 ± 0.05	6.08 ± 0.02	266.0 ± 5.60	1.17 ± 0.07	8.23 ± 0.02	18.98 ± 0.06	7.87 ± 0.07
NDA	29.23 ± 0.37	18.14 ± 0.06	2.38 ± 0.04	11.21 ± 0.01	318.5 ± 4.20	1.50 ± 0.05	8.31 ± 0.03	22.99 ± 0.06	7.88 ± 0.03
NDD	30.25 ± 0.81	18.66 ± 0.13	0.40 ± 0.02	11.82 ± 0.11	326.2 ± 0.46	1.08 ± 0.12	8.27 ± 0.03	28.28 ± 0.04	3.98 ± 0.06
NDE	25.95 ± 0.13	18.14 ± 0.08	1.39 ± 0.02	9.97 ± 0.05	293.3 ± 0.53	1.08 ± 0.09	8.22 ± 0.03	19.94 ± 0.11	3.63 ± 0.04
NDK	25.75 ± 0.11	18.79 ± 0.17	0.89 ± 0.03	6.12 ± 0.02	266.0 ± 17.58	0.50 ± 0.02	8.29 ± 0.03	15.90 ± 0.14	7.23 ± 0.02
NXF	32.50 ± 0.05	18.92 ± 0.09	0.40 ± 0.03	7.17 ± 0.03	302.4 ± 4.88	1.05 ± 0.04	8.29 ± 0.02	21.71 ± 0.07	7.97 ± 0.12
NXG	43.92 ± 0.10	20.22 ± 0.05	2.38 ± 0.10	11.24 ± 0.01	360.5 ± 2.25	8.71 ± 0.05	8.39 ± 0.02	24.97 ± 0.13	10.29 ± 0.05
FXF	32.90 ± 0.10	18.53 ± 0.10	0.89 ± 0.03	6.56 ± 0.02	308.0 ± 11.65	1.51 ± 0.08	5.76 ± 0.03	17.95 ± 0.07	6.04 ± 0.08
FXG	31.35 ± 0.06	16.97 ± 0.04	3.37 ± 0.04	4.75 ± 0.02	270.2 ± 4.48	0.63 ± 0.07	5.79 ± 0.04	15.57 ± 0.11	7.50 ± 0.07

### 2.5. Sequencing by synthesis

PCR used primers 341F-806R with the barcode was employed to amplify the variable region V3-4 of the 16S rRNA gene. The resulting amplicons were sequenced on the Illumina HiSeq2500 platform, and Novogene Bioinformatics Technology Co. (Beijing, China) generated 250 bp paired-end reads (Caporaso et al., 2011, 2012).

### 2.6. Sequence analysis

Paired-end reads were distributed to samples based on their unique barcode and merged by adopting FLASH (V1.2.7). The high-quality clean tags (Bokulich et al., 2013) were gained according to QIIME (V1.7.0) (Caporaso et al., 2010) quality-controlled process under specific filtering conditions. Effective tags were finally gained by filtering chimera sequences (Edgar et al., 2011). Uparse software (Uparse v7.0.1001) (Edgar, 2013) was employed to carry out sequence analyses, and taxonomic information was annotated by adopting GreenGene Database (DeSantis et al., 2006) based on the RDP classifier (version 2.2) (Wang et al., 2007). Sequences with at least 97% similarity were clustered into the same OTUs. MUSCLE software (version 3.8.3) was used to make multiple sequence alignments to research the phylogenetic relationships among different OTUs and the difference of the predominant species in different samples (Edgar, 2004).

### 2.7. Diversity and statistical analysis

The calculation of alpha-diversity measures, including the Shannon diversity index (Magurran, 1988), Chao1 richness estimator (Chao and Bunge, 2002), phylogenetic diversity (PD) index (Lozupone and Knight, 2008), and Good’s coverage (Good, 1953), was made. All indices were shown with R software (version 2.15.3) and calculated with QIIME (version 1.7.0). An unweighted pair group approach with arithmetic means (UPGMA) was carried out to cluster samples by weighted UniFrac distances (Johnson et al., 2003). Principal coordinates analysis (PCoA) applying unweighted UniFrac metrics was carried out to distinguish the distribution patterns of the bacterial community composition among the samples. The Mantel test and CCA were adopted to assess the linkages of the rhizosphere bacterial community structure with rhizosphere biogeochemical properties. CCA was conducted by applying functions in the Vegan package (version 2.3–0) of the R project (version 3.2.2).

### 2.8. Accession number(s)

The nucleotide sequences observed during this research have been stored in the NCBI database under the accession number SRP158785.

## 3. Results

### 3.1. General statistics analysis for 16S rRNA gene sequences and taxonomic compositions of the microbe communities

The total number of 16S rRNA tags gained from the 48 samples, after splicing, quality control, and filtering chimera, was 2,674,104, which were clustered into 163,219 operational taxonomic units (OTUs) with at least 97% sequence similarity in nucleotide identity, and those data were subjected for further statistical analyses (Table 3). The results suggested that Chao1 and ACE indices were between 2831.888–5271.669 and 2967.08–4390.37, respectively. It indicated a good richness of samples. Goods coverage was more than 0.955, indicating that the sequencing results could represent the sample microbial real situation.

**Table 3:** Mean bacteria diversity index of the samples.

Sample name	OTUs	Shannon	Simpson	Ace	Chao1 richness estimator	PD whole tree	Good’s coverage (%)
NWA	3311.33	9.27	0.9930	3347.80	3237.887	198.5860	97.13
NWB	3280.33	9.24	0.9943	3495.23	3392.368	191.6597	96.83
NWC	3108.00	9.18	0.9947	3141.87	3061.604	182.4097	97.27
NWK	3664.33	9.54	0.9947	4143.75	4072.341	222.0557	96.07
NPA	3464.00	9.65	0.9957	4390.37	5271.669	217.6163	95.73
NPD	3389.00	9.59	0.9957	3782.18	3718.123	209.1093	96.53
NPC	3265.00	9.45	0.9953	3283.17	3223.533	203.1617	97.17
NPK	3659.33	9.58	0.9953	3895.75	3755.389	215.9177	96.40
NDA	3362.67	9.69	0.9970	3511.51	3428.952	200.0757	97.00
NDD	3093.67	9.52	0.9967	3016.51	2996.649	183.8223	97.53
NDE	3216.33	9.64	0.9963	3459.30	3428.717	207.4817	97.00
NDK	3791.00	9.89	0.9970	3889.85	3804.239	215.0593	96.60
NXF	3709.67	9.71	0.9970	3978.43	3860.708	214.5317	96.30
NXG	3534.00	9.55	0.9960	3791.51	3708.465	202.2613	96.50
FXF	3728.00	9.16	0.9937	4090.29	3949.574	220.0440	96.07
FXG	2829.67	8.13	0.9877	2967.08	2831.888	166.9200	97.13

There were small gaps among rhizospheres microbial community richness index, ACE, and Chao1 of three plants under the same slope position(NWA, NWB, and NWC)in site A, while these indices of the same plant at different slope positions(NWA, NPA, and NDA)had a greater gap, but the indices of NPA were the highest. Shannon and Simpson diversity indices integrating evenness and species richness were comparable in NWA, NWB, and NWC. These two indices were higher in NDA and NPA than in NWA ones (Table 3). These results showed that slope positions could generate an effect on rhizosphere microbial diversity. By comparing the ACE and Chao1 indices of the same plant in the different soil types (NXF and FXF), we discovered that the indices of NXF were higher than those of FXF. In addition, the Shannon diversity indices of NXF (9.71) were higher than FXF (9.16), and the Shannon diversity indices of NXG (9.55) were greater than FXG (8.13). Such results indicated that soil types might have a significant relation with the rhizosphere microbial diversity.

**Figure 2:**
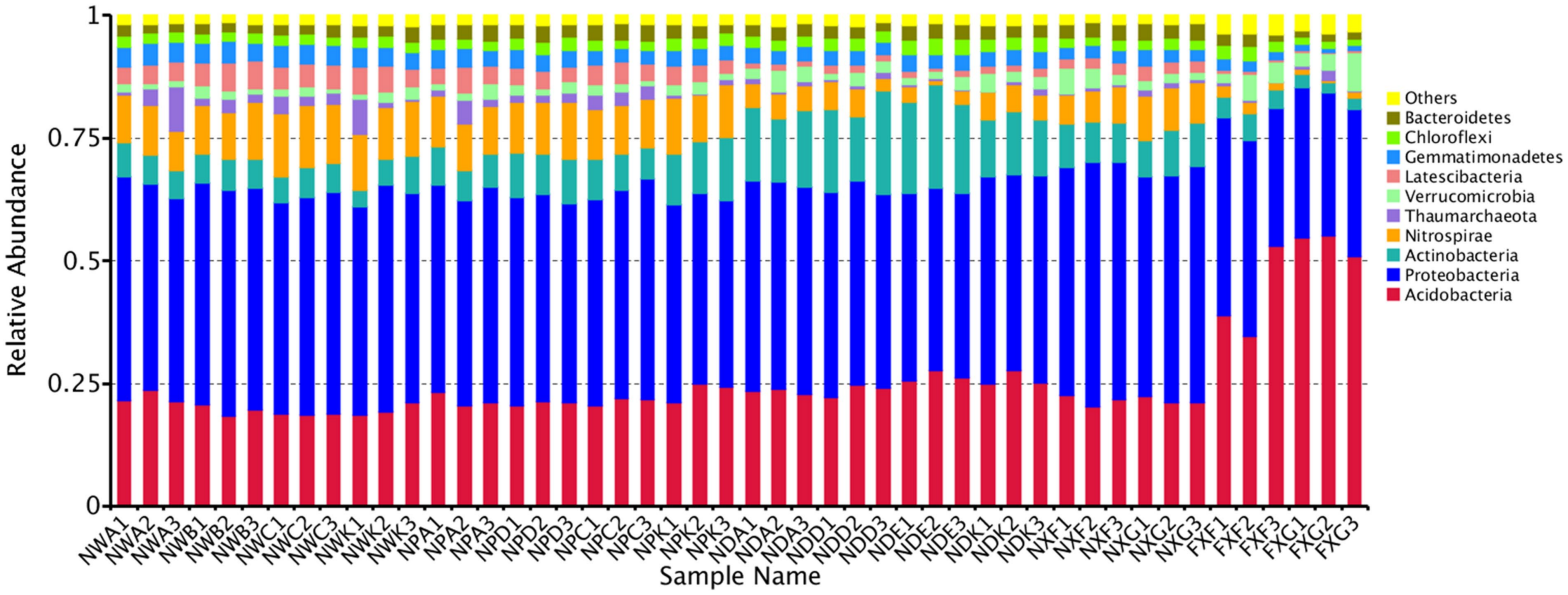
Microbe community structure is the 10 most abundant phyla in the 48 samples. All phyla were within the domain of *Bacteria*, except for the *Thaumarchaeota*, which was within the *Archaea*.

Operational taxonomic units were further classified into different taxa, and the estimation of their relative taxonomic abundance was made across different rhizospheres. A total of 100 bacteria classes were belonging to at least 48 phyla identified, including several unknown groups. The top 10 phyla with the highest relative abundance were *Acidobacteria, Proteobacteria, Actinobacteria, Nitrospirae, Thaumarchaeota, Verrucomicrobia, Latescibacteria, Gemmatimonadetes, Chloroflexi,* and *Bacteroidetes.* The *Proteobacteria* stood for the highest number of tags in rhizospheres and occupied at least 45.66% of the total bacteria population in samples, followed by *Acidobacteria* (34.21%), *Actinobacteria* (10.40%), and *Nitrospirae* (9.73%) (Figure 2). In the lime soil samples (NXF and NXG), *Proteobacteria* was the most abundant phylum with a content of 48.19 and 46.44%, respectively. In the laterite and brick red soil samples (FXF and FXG), *Acidobacteria* was the most abundant phylum with 42.12 and 53.58%, respectively. The foregoing results showed that there might be a causal relationship between various soil types with the dominant communities of rhizosphere microorganisms.

**Figure 3:**
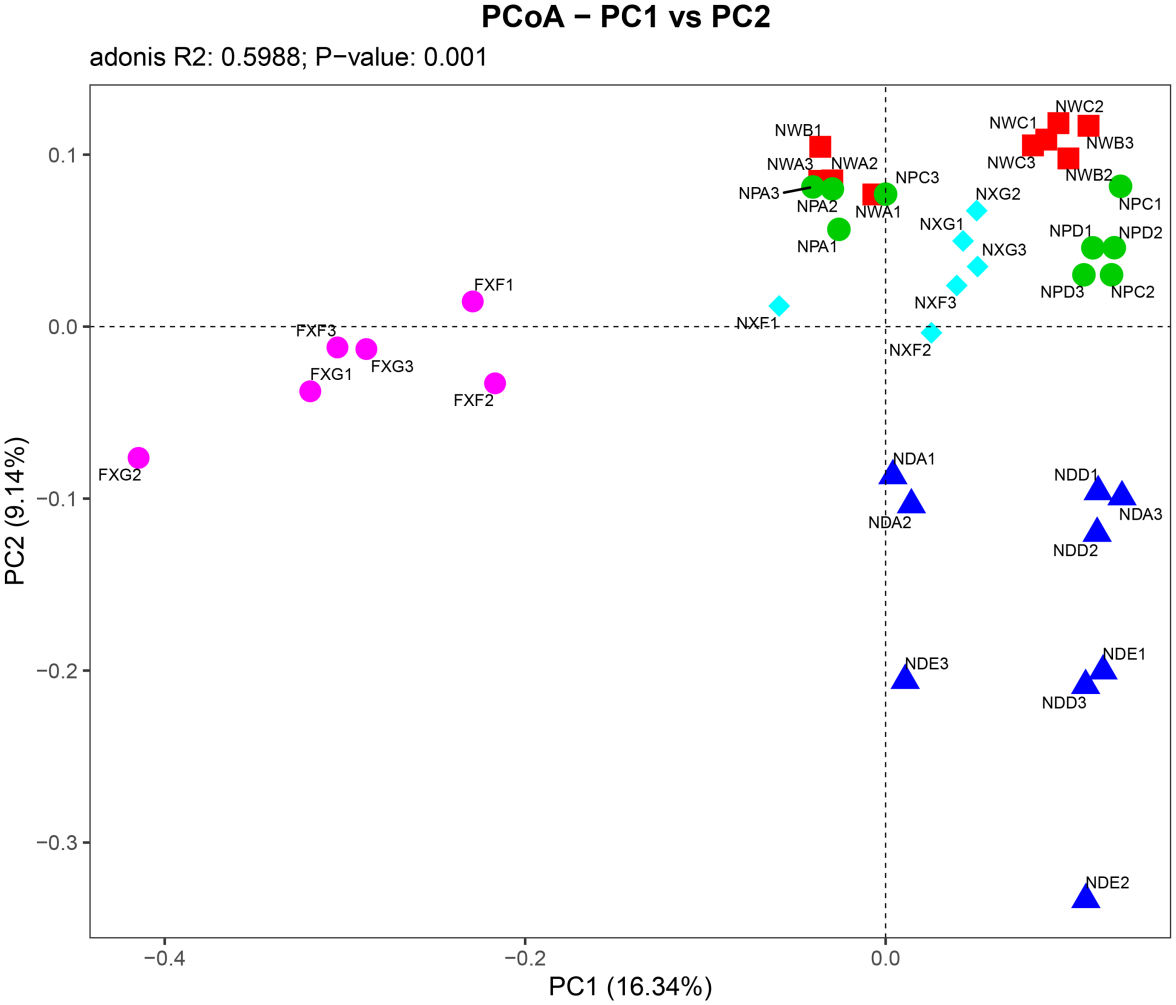
Associations between individual samples were explained by PCoA analysis. Symbols of the same color show sample from a similar habitat type and the distance between symbols display their dissimilarity. The detailed meaning of each sample was shown in Table 1, 1~3 represented three biological replicates.

**Figure 4:**
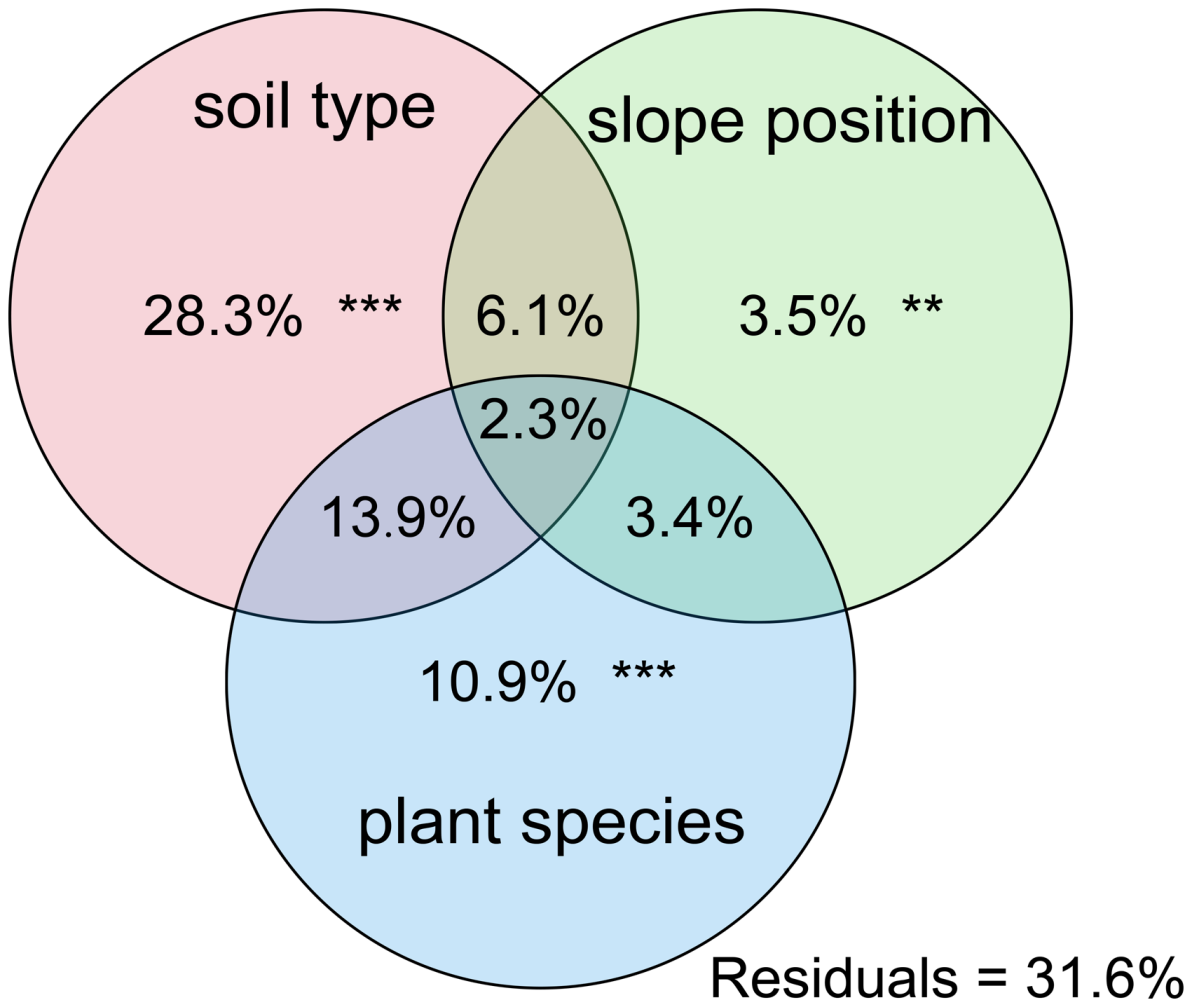
Contributions of soil type, slope position, and plant species on the assembly of soil bacterial communities were calculated based on variance partitioning analyses (VPAss), and *p*-value was determined by PERMANOVA.

Further PCoA analysis (Figure 3) was done based on OTUs, and it showed that microbial communities in the laterite and brick red soil samples (FXF and FXG) were separated from the cluster representing microbial communities of the lime soil samples (NXF and NXG). In addition, the bacteria community compositions in the toe slope of site A were similar to that of the back slope and these two were very different from the shoulder in the same soil type, and those resulting from the planting of different plant species in the same slope position were also significantly different (NPA, NPC, and NPD). PERMANOVA and VPA analyses showed that rhizosphere microbial communities were significantly affected by plant species (*p* < 0.001), slope position (*p* < 0.01), and soil type (p < 0.001), and that the relative importance of soil type on microbial communities was the greatest, followed by plant species and slope position (Figure 4).

### 3.2. LEfSe (LDA effect size) analysis of bacterial communities among plant species, slope positions, and soil types

**Figure 5:**
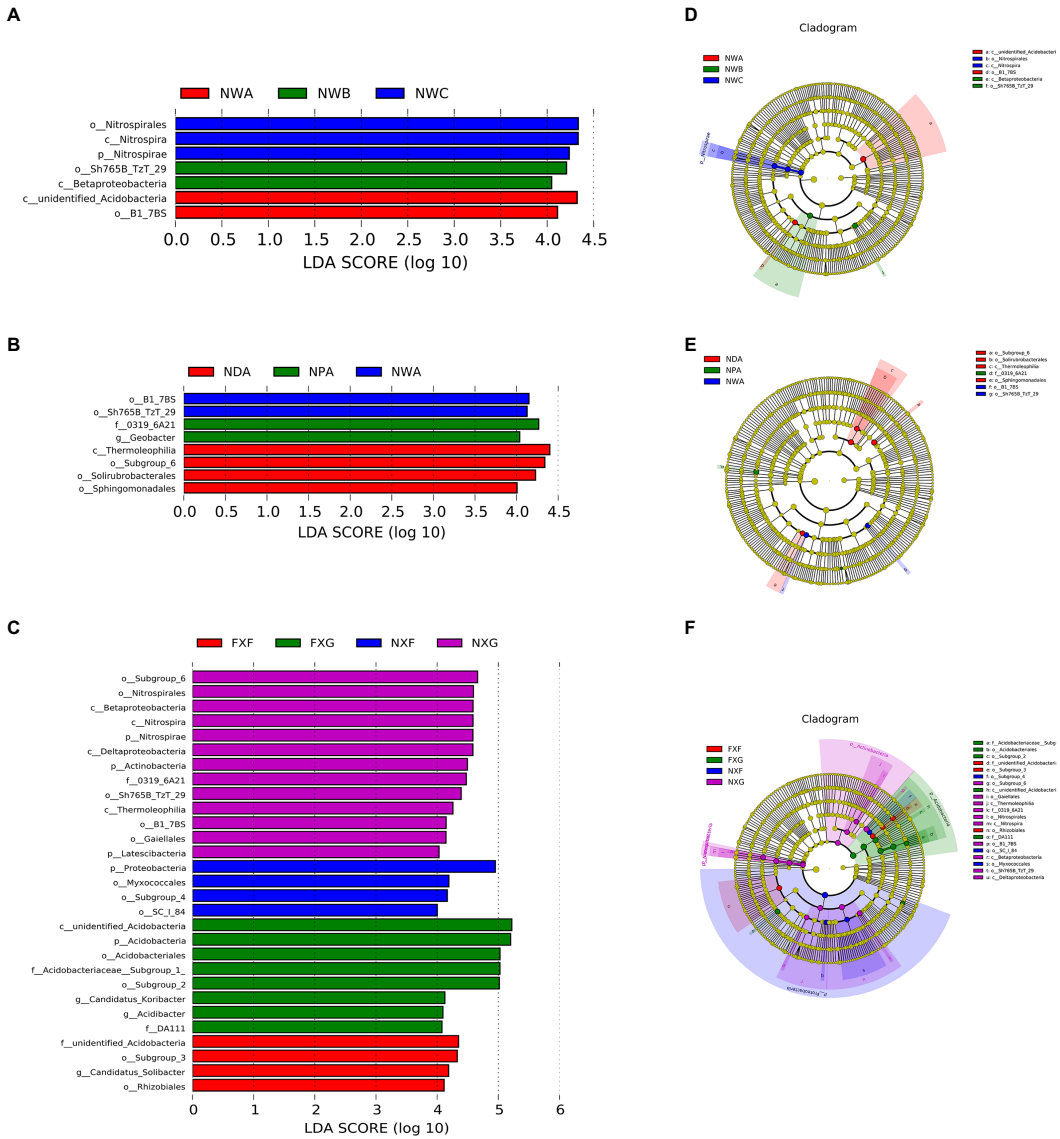
LDA value distribution histogram and evolutionary branches of LEfSe analysis. In the evolutionary cladogram, circles from inside to outside represented the classification level from the phylum to the species. Coloring principle: Species without great differences were uniformly colored yellow, and the Biomarker followed the group for coloration. The red node indicated that the microbial group played a significant role in the red group, and the green node indicated that the microbial group played a significant role in the green group. The blue node indicated that the microbial group played a significant role in the blue group, and the purple node indicated that the microbial group played a significant role in the purple group. **(A)**: LDA value distribution histogram of NWA, NWB, and NWC groups; **(B)**: LDA value distribution histogram of NDA, NPA, and NWA groups; **(C)**: LDA value distribution histogram of FXF, FXG, NXF, and NXG groups; **(D)**: Evolutionary cladogram of NWA, NWB, and NWC groups; **(E)**: Evolutionary cladogram of NDA, NPA, and NWA groups; **(F)**: Evolutionary cladogram of FXF, FXG, NXF, and NXG groups.

Linear discriminant analysis effect size was applied to the microbial data of the nine rhizosphere groups. Statistically different taxonomic clades with an LDA score higher than 4.0 (Figure 5) were found. The cladogram (Figures 5D–F) indicated that many taxa were common (shown in yellow), but some specific differences also existed.

The rhizosphere microbe of the *Sterculia monosperma*, *Saraca dives*, and *Catunaregam spinosa* (NWA, NWB, and NWC) were compared together to identify the impact of the different plant species in the same slope position and soil type on the bacterial community compositions (Figure 5A). The results suggested that the most differentially abundant bacterial taxa in these groups belonged to *unidentified_Acidobacteria*, *Betaproteobacteria*, and *Nitrospira* at the class level, respectively. At the order level, the enrichments of NWA, NWB, and NWC were *B1_7BS*, *Sh765B_TzT_29*, and *Nitrospirales*. It revealed that the influence of plant species on rhizosphere microbial community structure was still worth some attention.

The rhizosphere microbe of the *Sterculia monosperma* (NWA, NPA, and NDA) was compared together to identify the effect of the same plant species and soil type in different slope positions on the rhizosphere bacterial composition (Figure 5B). The outcomes revealed that the most differentially abundant bacterial taxa in NWA were *B1_7BS* and *Sh765B_TzT_29*, and in NDA were *Subgroup_6*, *Solirubrobacterales*, and *Sphingomonadales* at the order level. NPA enriched the family *0319_6A21* and the genus *Geobacter*. It indicated that slope positions might affect the rhizosphere microbial community structure of the same plant.

The rhizosphere microbe of the *Wendlandia uvariifolia* (NXF and FXF) and *Bridelia balansae* (NXG and FXG) in sites A and B were compared together to evaluate the difference in bacterial communities when the same species of plants lived in the different soil types (Figure 5C). NXF and FXF were located at different slope positions, while NXG and FXG were located at the same slope positions. The NXF rhizosphere generated a greatly higher relative abundance of the *Proteobacteria* phylum and *SC_I_84*, *Myxococcales*, and *Subgroup_4* orders, while the FXF rhizosphere had a higher relative abundance of the *Subgroup_3* and *Rhizobiales* orders, *unidentified_Acidobacteria* family, and *Candidatus_Solibacter* genus. The NXG rhizosphere enhanced the relative abundance of *Nitrospirae*, *Actinobacteria*, *Latescibacteria* Phyla, *Betaproteobacteria, Nitrospira*, *Deltaproteobacteria* and *Thermoleophilia* classes, *Subgroup_6*, *Nitrospirales*, *Sh765B_TzT_29*, *B1_7BS*, *Gaiellales* orders, and *0319_6A21* family. However, the FXG rhizosphere exhibited a higher relative abundance of the *Acidobacteria* phylum, the class *unidentified_Acidobacteria*, *Acidobacteriales*, and *Subgroup_2* orders, *DA111* and *Acidobacteriaceae__Subgroup_1* families, *Candidatus_Koribacter*, and *Acidibacter* genera. This result suggested that soil types could also affect the rhizosphere microbial community structure of the same plant.

### 3.3. CCA analysis of the biogeochemical properties of rhizosphere soil and bacterial community diversity

**Figure 6:**
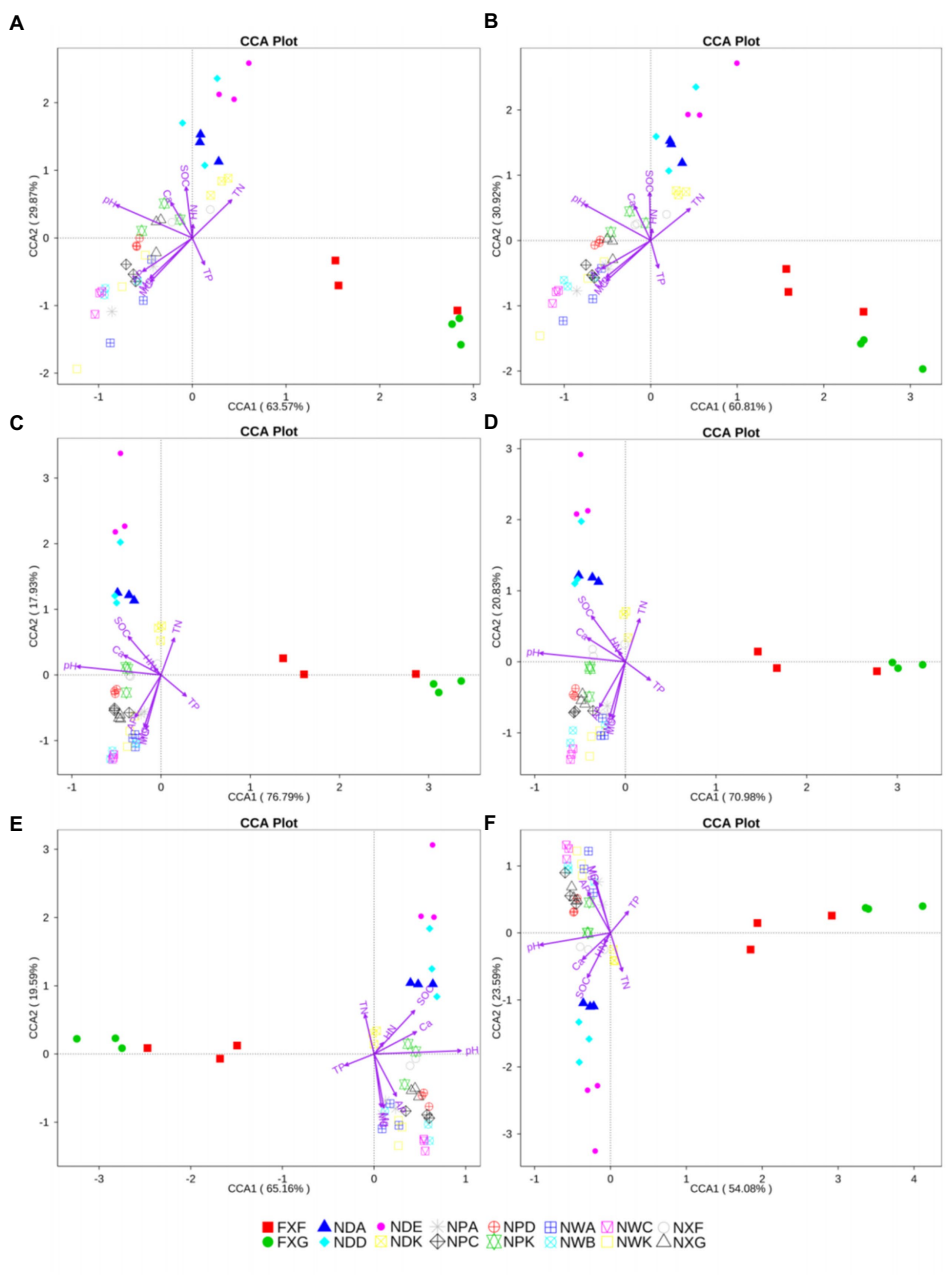
CCA analysis at the phylum **(A)**, class **(B)**, order **(C)**, family **(D)** genus **(E)**, and species **(F)** levels. The same symbols and colors indicated samples from the same group. Environmental parameters were demonstrated by purple arrows. The axes showed the percentages of variation in the distribution of microbe communities.

Nine variables, including pH, moisture content (MC), total nitrogen (TN), total phosphorus (TP), soil organic carbon (SOC), hydrolyzable nitrogen (HN), available phosphate (AP), calcium (Ca), and magnesium (Mg), were used in further CCA analysis. pH, MC, TN, TP, SOC, AP, Ca, and Mg were significant at all taxonomic levels (*p* < 0.05), except in the case of the TP at the genus level (*p* = 0.085). However, HN did not have a significant effect on the community structure (*p* > 0.05). HN was not a major factor for any of the groups at any taxonomic level (Figure 6). CCA of the relationships between the nine variables of biogeochemical properties in the rhizosphere and the bacteria community composition at different taxonomic levels revealed that community structures were most greatly influenced by pH. Among the explanatory variables, the order of influence was pH (*p* < 0.01) > MC (*p* < 0.01) > Mg (*p* < 0.01) > SOC (*p* < 0.01) (Figure 6).

## 4. Discussion

### 4.1. Plant species, slope positions, and soil types have different determinant roles in the rhizosphere microbial community structure

Under combined variations of plant species, slope positions, and soil types identity, the phenomenon was observed that the soil type was found to play a predominant role in the development of rhizosphere microbial communities (28.3% of separate contribution rate for bacteria), more than plant species identity (10.9% of separate contribution rate for bacteria) and slope position (3.5% of separate contribution rate for bacteria) (Figure 4). A few published reports have indicated that soil types have a greater effect on the root microbiota profiles than the plant species did (Bulgarelli et al., 2012; Lundberg et al., 2012; Veach et al., 2019). These reports were consistent with our results. However, many previous studies have shown that the influence of plant species on the rhizosphere microbial community is stronger than that of soil abiotic environment, or the influence of both is comparable. For example, Miethling et al. conducted experiments by collecting rhizosphere microorganisms of two plant species, alfalfa (Medicago sativa) and rye (Secale cereale), which grew on two kinds of soil collected from farmland in different locations, to compare the effects of plant species and soil types on plant root microbial communities. It was determined that plant species was the main determinant of microbial community characteristics, and soil was of minor importance (Miethling et al., 2000). Some scholars investigated the importance of pH, soil type, soil amendment, nutritional status of the plant, plant species, and plant age on the structure of the bacterial community in the rhizosphere by growing several kinds of different plant species (cucumber, barley, chickpea, canola, and Sudan grass) in three different soil types (sand, loam, and clay) and observed that many different factors will contribute to shaping the species composition in the rhizosphere, but that the plant itself exerts a highly selective effect that is at least as great as that of the soil(Marschner et al., 2004). Igwe and Vannette observed that plant species and soil type had comparable effects in structuring rhizoplane bacterial communities (Igwe and Vannette, 2019). Some researchers revealed that the soil type had a larger effect than plant species on fungal community composition, but the opposite was true for bacterial communities with a trap-plant bioassay experiment (Bonito et al., 2014). Our findings here are possibly due to (1) the influence of soil types on the rhizosphere microbial community structure under different geological backgrounds might be caused by the differences of nutrient elements, pH value, and other factors, which have more shaping effects than plants species and slope position; (2) different plants adaptively changed the composition of root exudates to absorb different microorganisms according to different slope environments, and the shaping effect was greater than that of slope environment (Figures 5A,B,D,E); and (3) although slope position has the least influence, it is worth noting that due to severe rain erosion and little soil retention, the rhizosphere microbial community compositions in the shoulder in karst areas were different from those of the back slope and toe slope (Figure 3).

### 4.2. The pH-based environmental factors were the main factors affecting the rhizosphere microbial community structure in the northern tropical seasonal rainforest

Microbial diversity analysis showed that the rhizosphere bacterial diversity was mostly affected by soil types. In further CCA analysis of rhizosphere soil biogeochemical properties and rhizosphere microbial community structure, pH was considered the most important variable factor governing the variation of the bacteria community structures in 48 samples gained from different types of northern tropical seasonal rainforests in China. Our results discovered great correlations between variable factors (pH, MC, Mg, and SOC) and the distributions of community composition (Figure 6). In those parameters, pH showed positive correlations with MC, Mg, and SOC across all samples. It was reported that microbial biogeography was controlled mainly by edaphic variables, and the biodiversity of soil bacterial communities differed by ecosystem type. Furthermore, these differences could largely be illustrated by soil pH (Fierer and Jackson, 2006), which produced a complicated effect on microbial communities by affecting the nutrients, microbial adsorption, etc. The pH value of the soil environment affected the migration and morphology of elements in the soil and was an important determinant of soil nutrient distribution.

Regarding microbial compositions of rhizospheres that developed in both sites, we found that the most dominant microbes showed significantly different relative abundances in the two soil types. This was observed for microbial phyla (Figure 2), such as *Acidobacteria, Proteobacteria, Actinobacteria, Nitrospirae, Thaumarchaeota, Verrucomicrobia, Latescibacteria,* and *Gemmatimonadetes*, indicating that these phyla were sensitive to the soil biogeochemical properties factors even if the same plant species. For example, the relative abundance of *Acidobacteria* doubled. Since the soil at site B was laterite and brick red soil (pH <7), the *Acidobacteria* could adapt to the foregoing acidic environment. However, the core bacterial species did not change much. *Proteobacteria*, *Acidobacteria*, and *Actinobacteria* still occupied the top three positions. It was similar to the rhizosphere microorganisms of sugar beet plants analyzed by PhiloChip, except that the dominant ones were *Proteobacteria*, *Firmicutes*, and *Actinobacteria* (Mendes et al., 2011), which confirmed our conclusion. Previous studies have suggested that soil abiotic environmental factors affected rhizosphere microorganisms, which might be due to differences in the nutrient distribution in different habitats (Shi et al., 2011). In short, these results indicate that different soil biogeochemical property factors have distinct effects on rhizosphere microbial community structures.

### 4.3. Some plants in the northern tropical seasonal rainforest might resist the adverse effects of nutrient tolerance and gain ecological advantages by coexisting with specific rhizosphere microorganisms

The rhizosphere microbial population of *Catunaregam spinosa* (NWC) in site A had its specific biomarker *Nitrospirae*. We noted that *Nitrospirae* could convert ammonia and nitrite into nitrates in nature that could be used directly by plants and provide nitrogen for plants (Attard et al., 2010; Daebeler et al., 2014). The reason for the earlier phenomenon might be due to the low MC in the shoulder (Table 2), in which the rainfall was particularly rapid (Liu et al., 2004; Shen et al., 2017), and the shrubs and herbaceous plants were scarce so that the consumption of nitrogen in the soil was less, and the high nitrogen content was sufficient for trees. Therefore, it was not necessary to additionally increase the supply of nitrogen by symbiosis with *Nitrospirae*. At the toe slope, this situation was just the opposite. Due to the high MC in the toe slope, the vegetation was rich, resulting in severe consumption of nitrogen (Table 2). In the absence of nitrogen, plants might form symbiotic relationships with *Nitrospirae* by providing them with carbon sources and others to obtain additional nitrogen and gain a competitive advantage. According to previous investigations, in the toe slope of the nitrogen deficiency, *Catunaregam spinosa* was, indeed, the dominant species (Huang et al., 2014). The earlier results suggested that the nitrogen-fixing or nitrogen-transforming ability of rhizosphere microorganisms was likely to be a decisive factor for plants to become dominant species in nitrogen-deficient habitats. The theory that microorganisms could co-produce nitrogen with plants and provide nitrogen to plants has been widely accepted (Delgado et al., 1998; Popescu, 1998). Our result revealed a new perspective that the formation of plant-dominant species might be related to rhizosphere microorganisms with nitrogen conversion ability. This phenomenon might due to the mutualism between plants and microorganisms. Plants could get benefits from mutualistic partnerships with a wide variety of bacteria, fungi, and animals. In the northern tropical seasonal rainforest, reciprocity with specific microorganisms provided benefits to plants such as nitrogen fixation, which might result in plant species being dominant. It also indicated that soil and hydrological changes that were associated with topography played a significant role in the habitat allocation of heterogeneous karst forests. The niche separation has major effects on keeping the diversity of this heterogeneous karst forest (Guo et al., 2017).

In addition, the earlier relationship might be dominated by plants. One of the rhizosphere biomarkers of the NWA group was *Sh765B_TzT_29*, and the *Geobacter* enriched in the NPA group, which belonged to the *Deltaproteobacteria*. It was reported that *Sh765B_TzT_29* could reduce the sulfur element in the zinc-rich arsenic environment (Baldwin et al., 2015), while *Geobacter* could reduce Fe^3+^ ions (Magnuson et al., 2000). Such microorganisms preferred an anaerobic environment and a lower redox potential. In the environment of shallow rhizosphere soil, it was hard to achieve such conditions. But the *Sterculia monosperma* might just be able to provide this environment, thus causing the *Sterculia monosperma rhizosphere* microorganisms to converge inhabit. It could be seen that the influence of plants on the rhizosphere environment might have a certain dominant position. Previous studies have shown that plants could regulate mycorrhizal fungi by regulating the supply of carbohydrates to resist nutritional constraints (Johnson et al., 2003). The results of this study suggested that there might be a similar mechanism that plants could regulate rhizosphere microbes.

In short, due to climate and topography, the soil nutrient distribution in the northern tropical seasonal rainforest was uneven. Plants might selectively adapt their rhizosphere microorganisms under such pressure to form symbiotic nitrogen fixation patterns and other possible ways to resist the lack of nutrients. This adaptation mechanism was likely to be the decisive factor in determining whether a plant could become a dominant species. Plants were likely to be the dominant players in this adaptation process. This relationship between plants and rhizosphere microorganisms could be used to help protect the diversity of karst forests.

## 5. Conclusion

Through the study, the factors influencing the development of rhizosphere microbial communities were explained in multiple scales. On the medium and small scale, plant species and slope position could explain part of it (site A). Meanwhile, on a large scale (sites A and B), it is mainly reflected in different soil types caused by different geological backgrounds (karst and non-karst). Overall, soil types had the greatest influence on rhizosphere microbial diversity, followed by plant species and finally slope positions. These differences could largely be illustrated by soil pH, which had a complicated effect on microbial communities by affecting the nutrients, microbial adsorption, and so on. In studying the plant diversity of the northern tropical seasonal rainforest, more attention should be paid to rhizosphere soil types, physicochemical properties, and rhizosphere microbial community. Moreover, plants might selectively adapt themselves to rhizosphere microorganisms *via* secretions and other ways to gain advantages of getting nitrogen and other characteristic nutrients. This adaptation mechanism would help the plants to use the resources and living space effectively and have great significance to ecological restoration and agricultural production.

## Data availability statement

The datasets presented in this study can be found in online repositories. The names of the repository/repositories and accession number(s) can be found below: https://www.ncbi.nlm.nih.gov/sra/SRP158785.

## Author contributions

XZ and XL: conceptualization and writing—original draft preparation. YG, BW, and TC: investigation. XZ, YG, BW, TC, and XL: writing—review and editing. XZ: visualization. XL: supervision. All authors have read and agreed to the published version of the manuscript.

## Funding

This research was supported by the National Natural Science Foundation of China (32271599, 32260276, and 31660130) and the Key Laboratory of Ecology of Rare and Endangered Species and Environmental Protection (Guangxi Normal University), Ministry of Education, China (ERESEP2021K03).

## Conflict of interest

The authors declare that the research was conducted in the absence of any commercial or financial relationships that could be construed as a potential conflict of interest.

## Publisher’s note

All claims expressed in this article are solely those of the authors and do not necessarily represent those of their affiliated organizations, or those of the publisher, the editors and the reviewers. Any product that may be evaluated in this article, or claim that may be made by its manufacturer, is not guaranteed or endorsed by the publisher.
